# Health and Social Inequalities in Women Living in Disadvantaged Conditions: A Focus on Gynecologic and Obstetric Health and Intimate Partner Violence

**DOI:** 10.1089/heq.2020.0133

**Published:** 2021-06-15

**Authors:** Massimo Ralli, Suleika Urbano, Elisabetta Gobbi, Nataliya Shkodina, Stefania Mariani, Aldo Morrone, Andrea Arcangeli, Lucia Ercoli

**Affiliations:** ^1^ Department of Sense Organs, Sapienza University of Rome, Rome, Italy.; ^2^ Primary Care Services, Eleemosynaria Apostolica, Vatican City State, Vatican City.; ^3^ Istituto di Medicina Solidale, Rome, Italy.; ^4^ San Gallicano Dermatological Institute, IRCCS, Rome, Italy.; ^5^ Directorate of Health and Hygiene, Vatican City State, Vatican City.; ^6^ Department of Anesthesiology, Intensive Care and Emergency Medicine, Fondazione Policlinico Universitario A. Gemelli IRCCS, Rome, Italy.; ^7^ Department of Biomedicine and Prevention, Tor Vergata University, Rome, Italy.

**Keywords:** health inequalities, intimate partner violence, poverty, screening, fragile populations

## Abstract

**Purpose:** Gynecologic and obstetric health and intimate partner violence are particularly influenced by social determinants of health, such as poverty, low education, and poor nutritional status, and by ethnic and racial factors. In this study, we evaluated health and social inequalities of women living in disadvantaged neighborhoods in the city of Rome, Italy.

**Methods:** The study included 128 women living in socioeconomically disadvantaged neighborhoods. For each woman, a medical record was compiled and a gynecologic examination with screening for cervical cancer was performed. Family network, risk factors for gender-based violence, and psychological abuse were also evaluated.

**Results:** The largest part of the sample, although had adequate schooling, was unemployed or had a low-status job; this was at the basis of intimate partner violence in about one-third of our sample. Nearly 35% of our sample was composed of pregnant women; about half of them were not assisted by the public health system for routine obstetric examinations. Common findings at gynecologic examination for nonpregnant women were infections (*n*=18, 19.9%), pregnancy planning (*n*=13, 13.7%), menopause management (*n*=12, 12.6%), ovarian fibromas (*n*=6, 6.3%), and post-partum assistance (*n*=3, 3.2%). Screening for cervical cancer was executed in 62 women; 9 (14.5%) had low- or high-grade squamous intraepithelial lesion or cervical carcinoma.

**Conclusions:** Health and social inequalities are frequent in women living in disadvantaged conditions, with serious consequences for health and quality of life of women and of their children. Prevention and treatment, especially for the most vulnerable subjects, should be a priority for the public health system.

## Introduction

Gender differences have a significant impact on physical, psychological, and socioeconomic status.^1,2^ Women have higher chances to get sick and consume more drugs, and are socially disadvantaged compared to men for physical and psychological violence, higher unemployment rates, and economic freedom.^1–4^ In the United States, women's poverty rates are substantially above the rates for men, with a poverty rate of 14.5% for women and 11% for men, and nearly 18 million women living in poverty.^5,6^ Similar rates have been reported in Europe.^7,8^

Living in disadvantaged neighborhoods may further increase the gender gap, as groups of population with socioeconomic disadvantages are more likely to have bad health conditions and suffer more from chronic diseases; in addition, they have less chances to receive proper health assistance and develop more frequently acute and chronic diseases.^9–11^

**FIG. 1.**
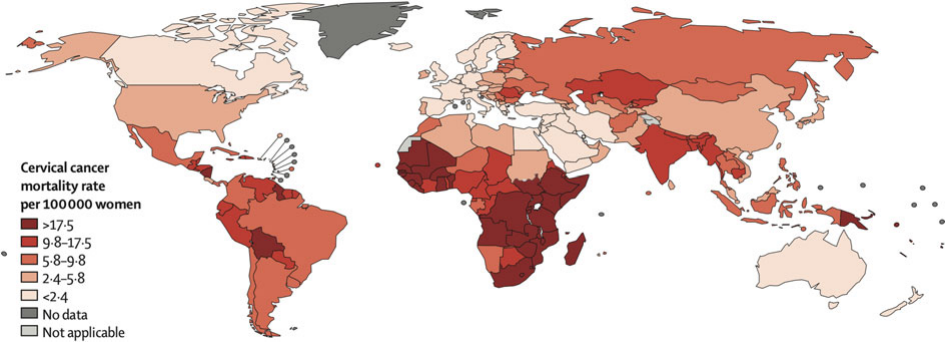
Global map of the age-standardized (world) mortality rates of cervical cancer in 2012, with the range divided into quintiles. From Ginsburg et al.^14^

Gynecologic and obstetric health of women is strongly influenced by social determinants of health, such as poverty, low education, and poor nutritional status, and by ethnic and racial factors.^12–15^ Evidence has shown that women with lower socioeconomic status have significantly higher incidence of benign and malignant gynecologic conditions compared to those with higher socioeconomic status, regardless of demographic factors such as race and ethnicity.^16^ Approximately 85% of women diagnosed and nearly 90% of women who die from cervical cancer live in a low-to-middle-income country; this is mainly due to missing prevention and limited treatment options (Fig. 1).^14^ At the same time, studies have shown that infant and maternal mortality rates during delivery are up to three times higher in black women compared to white women.^16^

Intimate partner violence is a global health issue regardless of demographic, ethnic, cultural, and socioeconomic conditions.^17^ However, studies have demonstrated that poor socioeconomic status may increase the risk of intimate partner violence, especially for young women in families with a yearly income inferior to $10,000.^17^ Research has shown that separated women and those with unemployed partners and low household income experience more abuse. In addition, pregnancy can represent a period of specific vulnerability to partner violence due to changes in women's physical, social, emotional, and financial needs, making women at the highest risk for intimate partner violence during this time.^18^

In this study, we evaluated several key elements of women living in socioeconomically disadvantaged neighborhoods in the city of Rome, Italy, with special focus on gynecologic and obstetric health and intimate partner violence.

## Methods

The study was performed between September 2019 and August 2020 and included 128 women living in socioeconomically disadvantaged neighborhoods referring to two primary care services. The first was the Madre di Misericordia Primary Care Center of the Eleemosynaria Apostolica, Holy See, located in the Vatican City State and its mobile health care facilities; and the second was the Medicina Solidale Center, located in a suburban area of the city of Rome, Italy.

For each woman, a general medical record was compiled to collect personal data, medical history, conditions of vulnerability with respect to schooling, access to health care services, housing, work, and the availability of a family network.

A psychological interview aimed at evaluating the presence of a family network and its socioeconomic status, detecting risk factors for gender-based violence and psychological abuse, was performed.

A gynecologic and obstetric record was also filled for each patient with the following information: age of the menarche, menopause, trend of menstrual cycles, family or personal history for breast, uterus, and ovary cancer, and current medical conditions with special attention to gynecologic issues. All women underwent a general gynecologic examination; screening for neoplastic pathology of the uterine cervix through the execution of a Pap test was performed in a portion of them.

The study was conducted in accordance with the Declaration of Helsinki; all patients signed a written informed consent to participate to the study. The study was reviewed by the board of the Istituto di Medicina Solidale association, which specifically approved it.

## Results

### Demographic and socioeconomic characteristics

**FIG. 2.**
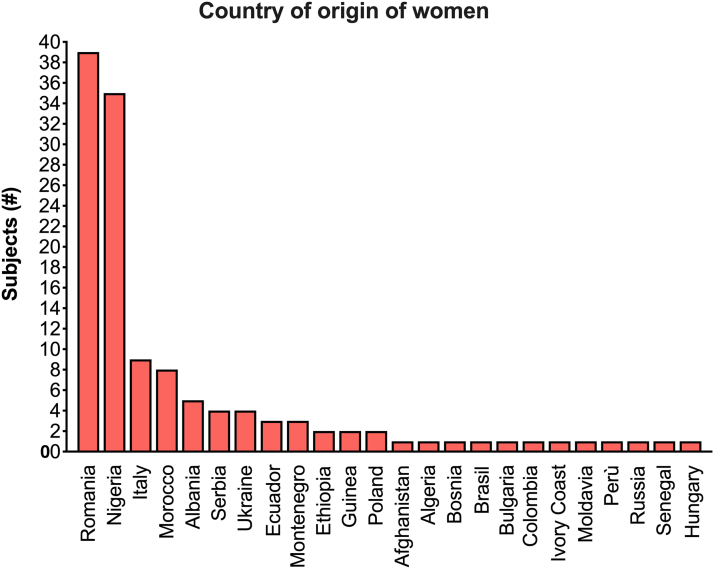
Country of origin of women included in the study.

**FIG. 3.**
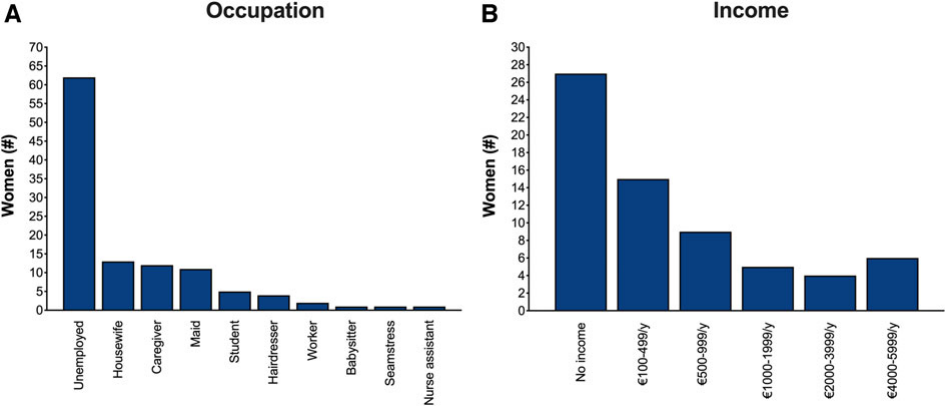
Occupation **(A)** and income **(B)** of women included in the sample.

The study included 128 women from 24 different countries; the most represented were Romania and Nigeria (Fig. 2). Average age was 38.1 years (18–72 years); the majority of women were married (*n*=68, 53.1%), had more than one child (*n*=76, 59.4%), high school degree (*n*=59, 46.1%), and were of Catholic religion (*n*=72, 56.3%) (Table 1). Nearly half of the sample were unemployed (*n*=61, 47.7%); the remaining 52.3% were employed (Fig. 3A). Over 20% of the women had no income, while about 18% had a yearly income lower than €1000 (Fig. 3B).

**Table 1.** Demographic Characteristics of Our Sample

Marital status		
Married	68	53.1%
Single	42	32.8%
Cohabitation	11	8.6%
Divorced	5	3.9%
Widow	1	0.8%
Marital separation	1	0.8%
Children		
0	28	21.9%
1	25	19.5%
>1	75	58.6%
Education		
Primary school	15	11.7%
Middle school	42	32.8%
High school	59	46.1%
University	9	7%
Unschooled	3	2.3%
Religion		
Catholic	72	56.3%
Orthodox	42	32.8%
Muslim	9	7%
Evangelical	2	1.6%
Pentecostal	3	2.3%

### Family network and intimate partner violence

The presence of a family network, its socioeconomic status, and potential intimate partner violence were evaluated through an interview with a psychologist.

The majority of women enrolled in the study had no family network, with all relatives abroad (*n*=74, 57.8%), while the remaining had at least one relative (*n*=28, 21.9%) or the entire family (*n*=26, 20.3%) in the same city. Nineteen women (14.8%) had at least one child in the country of origin.

Recurrent conflicts with partner were reported by 37 women (28.9%); the main reasons were economic issues mainly due to precarious housing and working conditions of the family. Frequent intimate partner violence episodes were reported by eight women (6.2%).

### Gynecologic and obstetric health status evaluation

Among the study sample, 33 women were pregnant, while 95 were not. The most common reason for the visit for nonpregnant women was gynecologic infections (*n*=18, 19.9%), pregnancy planning (*n*=13, 13.7%), menopause management (*n*=12, 12.6%), contraception (*n*=9, 9.5%), ovarian fibromas (*n*=6, 6.3%), and post-partum assistance (*n*=3, 3.2%). Adequate medical or surgical treatment was proposed.

The majority of enrolled patients had formal access to the public health system, although in some cases without economic exemption (*n*=88, 68.7%); however, more than half (*n*=83, 64.8%) reported that they had not requested public assistance in the last year due to financial or administrative issues.

### Screening for cervical cancer

Screening for cervical cancer was executed through the pap test; nearly 53% of the sample (67 women) never executed the screening procedure in the past. Pap test was performed in 62/128 women (48.4%); results were negative in 69.4% of cases, while revealed low-grade squamous intraepithelial lesions (SIL) in 5 women (8.1%), and high-grade SIL in 2 women (3.2%). Two women (3.2%) had advanced human papilloma virus (HPV)-related cervical cancer at the time of the screening. Infectious conditions were found in 10 women (16.4%); bacterial in 80% of cases; and mycotic in 20%. Women with SIL were addressed to public health structures for monitoring and intervention; women with infectious conditions were treated with appropriate therapy.

### Pregnancy

Thirty-three women were pregnant at the time of inclusion in the study. Twelve (36.4%) were in the first trimester, 10 (30.3%) in the second, and 11 (33.3%) in the third. Scheduled blood tests, including TORCH panel and HIV, Treponema pallidum Hemagglutination Assay, and Venereal Disease Research Laboratory screen and ultrasound in the public health system, were performed in, respectively, 12 (36.4%) and 26 (78.8%) women.

Ultrasound was negative for pathologic conditions of fetus in all cases; results of the blood tests are detailed in Figure 2.

## Discussion

Our study allowed a precise evaluation of demographic characteristics, availability of a family network, intimate partner violence, gynecologic and obstetric health, and access to public health care system of women living in disadvantaged socioeconomic conditions. This evaluation was performed through primary care services consisting in clinics and mobile health care facilities.

Socioeconomic analysis showed that the largest part of the sample, although had adequate schooling confirmed by high school or university degree in 53.1% of cases, were unemployed or had a low-status job. Literature has shown that women with low-status jobs and no decision-making authority have higher levels of negative life events and insecure housing tenure, and experience chronic stressors and reduced social support.^19–21^ Men are paid on average more than women, even in the case of similar levels of education and fields of occupation.^22–23^ Reduced income, coupled with longer life expectancy and increased responsibility to raise children, increases probabilities that women will face economic disadvantages. This may lead to reduced quality of life with higher rates of depression and anxiety, especially among poor women and in developing countries.^19–21^ Furthermore, women with low income are more likely to develop alcohol and drug addiction, which are significantly influenced by the social stressors linked to poverty.^24^

Economic issues were also at the basis of familial conflicts and intimate partner violence, as confirmed by nearly one-third of the sample. Partner violence has severe consequences on health and on other aspects of human life,^25^ such as chronic pain, physical disability, substance abuse and depression, and sexual and reproductive health complications, including sexually transmitted infections.^26^ This can contribute to worsen gender disparities.^27^

Poverty also influences physical conditions of women. Women in disadvantaged socioeconomic conditions have more chances to die for cancer than the general population for a lack of screening, prevention, and treatment.^28^ Obesity, risk for becoming obese, and staying obese from adolescence to young adulthood are strongly related to poverty among women.^29–31^ Also, infectious disease such as HIV and hepatitis C virus are more common among disadvantaged women.^32^

In our sample, over half of the women reported limited access to public structures due to financial or administrative issues. This applied both to pregnant and nonpregnant women, and to screening procedures. For the latter, more than half of our sample never performed a pap test and therefore never underwent gynecologic screening procedures. When performing pap test in our sample, we found 9 patients with low- or high-grade SIL or advanced HPV-related cervical cancer; this demonstrates a high rate of positive pap test at a screening level (14.5%), further confirming the potentially dramatic effects of missed screening and health care assistance in disadvantaged women.

Limited penetration of screening procedures and low access to public health care system for disadvantaged women, although interconnected, may represent a serious issue for public health, as may increase chances of disease progression, delayed diagnosis especially for cancer, and chronicization of diseases and permanent disabilities for women and their children, especially for pregnant women, which may not follow the scheduled examinations during pregnancy.

This study has several limits. The first is the small size of the sample, followed by the absence in the results of some anamnestic and clinical data collected during the initial and follow-up visits. Last, the sample was recruited in two primary care services and cannot be considered representative of all disadvantaged neighborhoods of the city, as it may reflect specific peculiarities of the neighborhoods where the primary care services were located.

## Conclusions

Most of the women enrolled in the study had difficulties in accessing public health system for cancer screening, disease treatment, and pregnancy monitoring. This may result in serious consequences for health and quality of life of women and of their children. In addition, a significant percentage of women reported intimate partner violence episodes and absence of a familial support network; these conditions are particularly evident in disadvantaged socioeconomic situations that, in most cases, are at the basis of these episodes. Gender differences have a significant impact on physical and psychological health, and disadvantaged socioeconomic conditions may further worsen it. Prevention and treatment, especially for the most vulnerable subjects, should be a priority for the public health and social system.

## Abbreviations Used

HPV: human papilloma virus
SIL: squamous intraepithelial lesions
